# Supplementary material for: Data Processing of SPR Curve Data to Maximize the Extraction of Changes in Electrochemical SPR Measurements
Source: Biosensors (Basel). 2022 Aug 8;12(8):615. doi: 10.3390/bios12080615 (PMC9406148; doi:10.3390/bios12080615)
Supplement: Supplementary file 1 [file biosensors-12-00615-s001.zip › biosensors-1804076-supplementary.pdf]

Supplemental

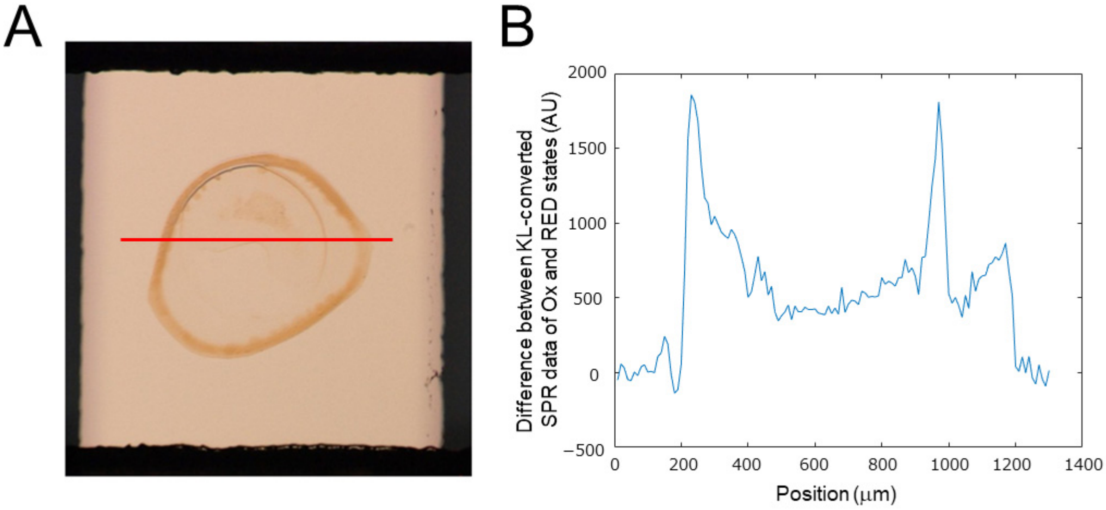

Figure S1. Evaluation of the distribution of changes in the SPR curve. A) Photograph of the osmium polymer film. B) Difference between KL-converted SPR curves in the fully oxidized and fully reduced states at SPR observation position of the red line in (A).

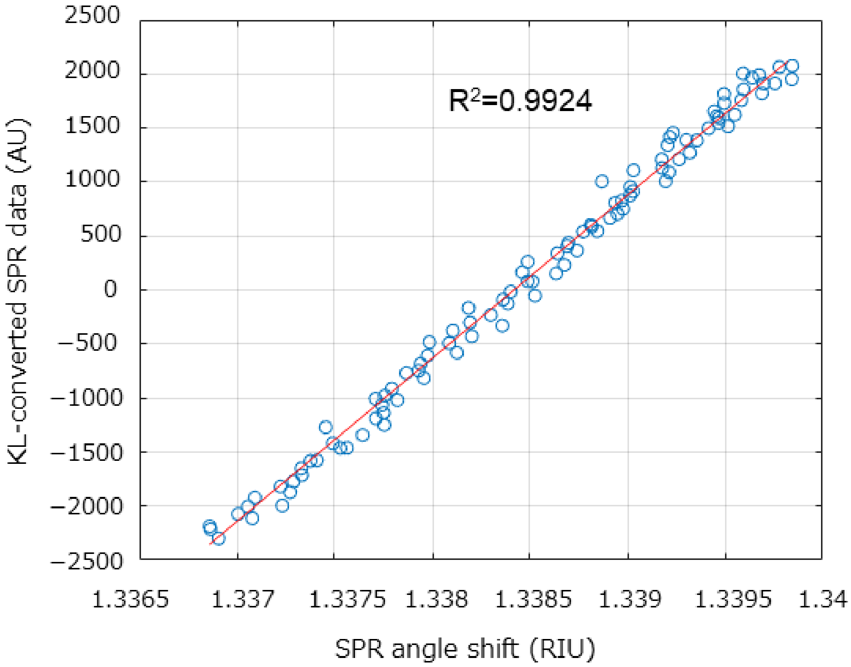

Figure S2. Correlation between standard detection of SPR angle shift and KL-converted SPR curve data when measuring SPR in NaCl solution.

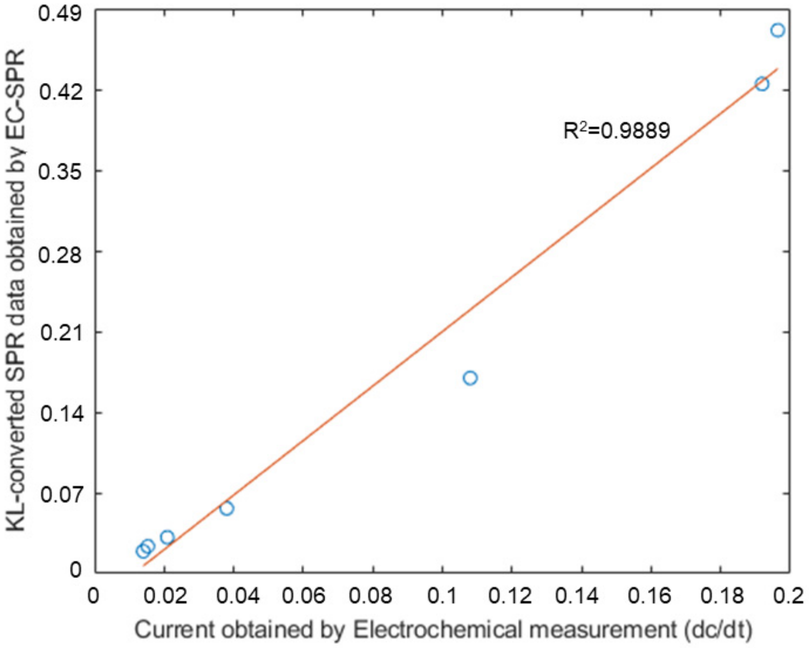

Figure S3. Correlation between general electrochemical measurement and KL-converted SPR curve data obtained from EC-SPR measurement. Red line: Regression line, R2: the coefficient of determination calculated from the correlation coefficient.

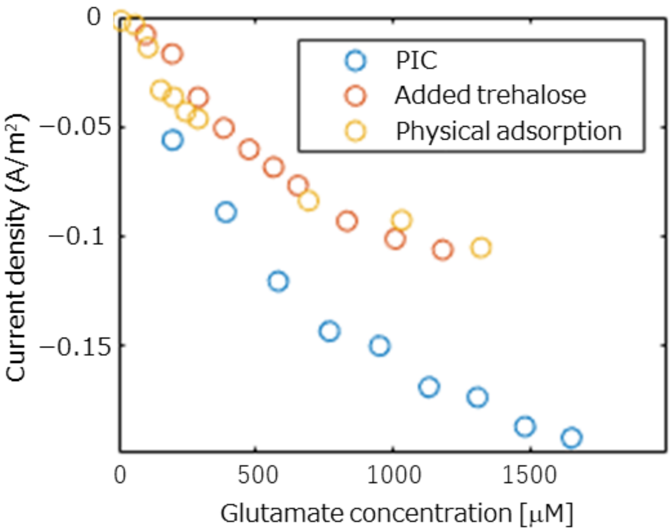

Figure S4. Comparison of electrochemical measurements by enzyme immobilization method. PIC: Poly-ion-complex by using gellan gum and Poly-L-lysine.

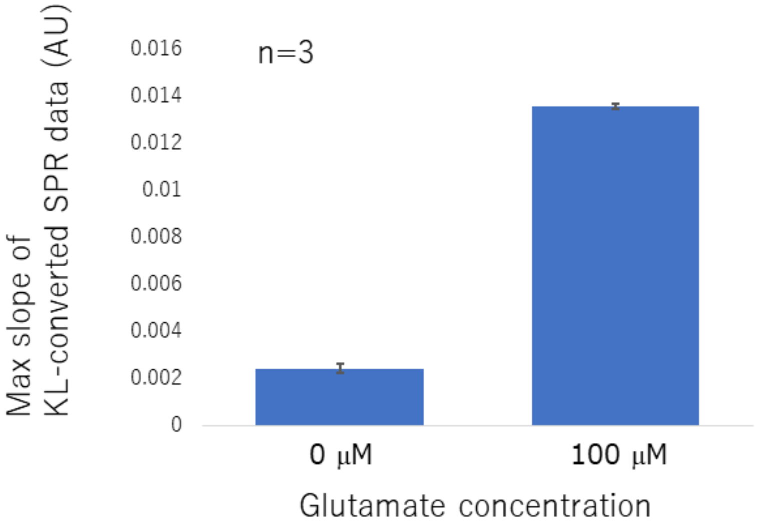

Figure S5. The reproducibility of the EC-SPR measurements of glutamate detection.
